# Development and Validation of CKD‐MKAP: A Malnutrition Perceived Knowledge, Attitude, and Practice (KAP) Scale for Patients With Chronic Kidney Diseases (CKD) in China

**DOI:** 10.1002/nop2.70925

**Published:** 2026-10-09

**Authors:** Shengrui Zhu, Hafzan Yusoff, Kueh Yee Cheng, Dariah Mohd Yusoff, Xiujuan Feng, Wen‐Kai Zheng, Hongfang Chen

**Affiliations:** ^1^ School of Medicine Xizang Minzu University Xianyang People's Republic of China; ^2^ Nutrition and Dietetics Programme, School of Health Sciences, Health Campus Universiti Sains Malaysia Kubang Kerian Malaysia; ^3^ Biostatistics and Research Methodology Unit, School of Medical Sciences, Health Campus Universiti Sains Malaysia Kubang Kerian Malaysia; ^4^ Nursing Programme, School of Health Sciences, Health Campus Universiti Sains Malaysia Kubang Kerian Malaysia; ^5^ School of Basic Medicine Inner Mongolia Medical University Hohhot People's Republic of China; ^6^ Human Resources Office Xi'an Provincial Hospital of Traditional Chinese Medicine Xi'an People's Republic of China

**Keywords:** attitude, chronic kidney disease, knowledge, malnutrition, practice

## Abstract

**Aim:**

This study aimed to develop and validate a culturally tailored scale (CKD‐MKAP) for assessing malnutrition‐related perceived knowledge, attitude and practice (KAP) among patients with chronic kidney disease (CKD) in China.

**Methods:**

The initial item pool was developed through a comprehensive literature review, semi‐structured interviews and the Delphi method. Content validity was evaluated by an experts panel. Construct validity was initially explored using exploratory factor analysis (EFA) and subsequently verified via confirmatory factor analysis (CFA).

**Results:**

During the development phase, a 27‐item scale was established following content validity assessment and a pilot study. Subsequent item analysis of the ‘Perceived Knowledge (K)’ domain led to the deletion of item K1. EFA (*n* = 152) for the ‘Attitude (A)’ and ‘Practice (P)’ domains yielded factor loadings exceeding 0.50, explaining 69.87% and 61.84% of the total variance, respectively. CFA (*n* = 151) demonstrated that the final 6‐item model for the ‘K’ domain exhibited a good model fit: RMSEA (90% CI) = 0.070 (0.000–0.124), CFI = 0.978, TLI = 0.963 and SRMR = 0.061. Similarly, the 9‐item ‘A’ domain and the 10‐item ‘P’ domain exhibited excellent fit indices. The three domains demonstrated satisfactory to excellent internal consistency, with Cronbach's alpha coefficients of 0.740 for Perceived Knowledge, 0.970 for Attitude and 0.958 for Practice.

**Conclusion:**

The CKD‐MKAP is a valid and reliable instrument that can be effectively utilized by clinical nurses to evaluate the malnutrition‐related KAP among CKD patients in mainland China.

**Implications for the Profession and/or Patient Care:**

The CKD‐MKAP scale offers healthcare professionals a valid, culturally tailored and time‐efficient tool to assess malnutrition‐related cognition and behaviours in CKD patients. Identifying specific deficits in perceived knowledge, attitudes or practices allows for personalized nutritional interventions, optimizing patient care and self‐management.

**Impact:**

This study addresses a critical gap in CKD management in China by introducing a multidimensional nutritional KAP assessment tool. The application of this scale can facilitate the early identification of malnutrition risks, improve the precision of patient education and ultimately contribute to slowing CKD progression and reducing the disease burden associated with malnutrition.

**Reporting Method:**

The methodology and findings of this multiphase quantitative study are reported in accordance with standard psychometric validation procedures.

**Patient or Public Contribution:**

Patients' lived experiences, shared during qualitative interviews, were used to generate the initial item pool and ensure the scale reflected patient perspectives and cultural nuances.

## 1 Introduction

Chronic kidney disease (CKD) affects approximately 10% of the global adult population, constituting a rapidly growing public health burden (Bikbov et al. 2020). Over the past two decades, CKD has risen from 27th to 18th leading cause of death globally (Liyanage et al. 2015). In China, the prevalence is particularly high at 13.2%, with substantial regional disparities (Lv and Zhang 2019). Given the substantial economic strain associated with CKD progression and dialysis (Webster et al. 2017), effective disease management has become a critical epidemiological challenge, particularly in low‐ and middle‐income settings (Rovin et al. 2021).

Patients with CKD are highly susceptible to malnutrition, a condition driven by a multifactorial aetiology that includes reduced nutrient intake and metabolic derangements (Iorember 2018). Malnutrition in this population often manifests as protein‐energy wasting, characterized by the loss of muscle mass, decreased physical functionality and unintentional weight loss (Becker et al. 2014; Fahal 2014; Hyun et al. 2017). The prevalence of malnutrition increases markedly as renal function declines, ranging from 11% to 54% in CKD Stages 3 to 5 (Carrero et al. 2018) and often exceeding 50% in Stage 5 patients prior to dialysis initiation (Hanna et al. 2020). Surveys conducted in China report similar prevalence rates ranging from 20% to 50% (Song et al. 2018). Crucially, malnutrition serves as a major catalyst for the adverse progression of CKD, acting as a primary predictor of increased hospitalization, morbidity and mortality (Iorember 2018).

While physiological and demographic variables (e.g., age, comorbidities, CKD stage) contribute to malnutrition, dietary behaviours and self‐management capabilities remain highly modifiable risk factors (Azzeh et al. 2022; Hyun et al. 2017; Xi et al. 2023). Within this context, individual cognitive and behavioural determinants—specifically perceived knowledge, attitudes and practices (KAP)—play an indispensable role in nutritional management (Vázquez‐Espino et al. 2020). According to the KAP behavioural change theory, acquiring accurate nutritional perceived knowledge fosters positive attitudes, which subsequently translate into effective dietary practices and lifestyle modifications (Onbe and Kanda 2018). Therefore, systematically assessing the malnutrition‐ related KAP of CKD patients is essential for identifying risk profiles and may hold promise for evaluating the efficacy of nutritional intervention programs, pending further evaluation of the tool's responsiveness to change.

Despite this clinical need, there is a notable lack of culturally appropriate and rigorously validated instruments to assess malnutrition‐related KAP among CKD patients in China. Existing tools in China largely focus on isolated dimensions of KAP rather than providing a comprehensive assessment (Chen et al. 2019; Li 2020; Zou 2017). Furthermore, while one comprehensive questionnaire has been proposed, it lacks robust psychometric validation (Zhang et al. 2021). Additionally, previously developed international scales (Onbe and Kanda 2018; Vlaminck et al. 2001) are heavily influenced by their specific socio‐economic and cultural environments, limiting their cross‐cultural applicability to the Chinese healthcare context. To bridge this research gap, a comprehensive, culturally relevant and scientifically validated evaluation tool is urgently required. Therefore, the present study aimed to develop a specialized malnutrition‐related KAP scale for CKD patients (CKD‐MKAP) in China and comprehensively evaluate its psychometric properties using exploratory factor analysis (EFA) and confirmatory factor analysis (CFA).

## 2 Methods

### 2.1 Scale Development

This study employed a multiphase quantitative design for scale development. It is important to note that the initial qualitative phase (comprehensive literature review and semi‐structured interviews with 13 patients) has been previously published (Zhu, Gao, et al. 2024). While the previous publication detailed the thematic findings regarding patients' perceptions, the current manuscript distinguishes itself by focusing entirely on the new instrument‐development process and the rigorous psychometric validation of the quantitative CKD‐MKAP scale. During the item generation phase, the initial item pool was constructed by translating the published qualitative themes and literature review findings into measurable quantitative constructs.

#### 2.1.1 Creation of the Initial Item Pool

Initially, a comprehensive literature review was conducted focusing on the Knowledge‐Attitude‐Practice (KAP) framework and relevant clinical guidelines, including those for nutrition in CKD (Ikizler et al. 2020) and undernutrition in CKD (Wright et al. 2019).

Subsequently, localized perceptions of malnutrition‐related KAP were explored through qualitative interviews with 13 CKD patients with malnutrition, using the interview guide presented in Table 1. The detailed methodology regarding patient recruitment dates, interview counts, data saturation and deductive content analysis has been fully described in our previous qualitative publication (Zhu, Mohd Yusoff, et al. 2024). The qualitative findings served as a foundational formative phase. By synthesizing these previously published lived experiences with the literature review, we successfully mapped the qualitative themes onto the KAP framework, generating an initial pool of 38 items.

**TABLE 1:** Interview guide items.

No. of item	Question
Question 1	What do you know about malnutrition in chronic kidney disease? Have you received any health education on appropriate nutrition for your disease? Are you satisfied with the information provided?
Question 2	Do you think that you are now malnourished? If yes, why do you think so?
Question 3	When exactly did you develop malnutrition?
Question 4	How do you feel when you are malnourished?
Question 5	What factors do you think may contribute to malnutrition? and why?
Question 6	Do you think it is dangerous? If yes, how does this condition (malnourished) affect your daily life or health?
Question 7	Are you satisfied with your current nutritional status? What are your thoughts or opinions on your diet and nutrition?
Question 8	Do you think malnutrition can be prevented and how did you prevent the occurrence of malnutrition? And why?
Question 9	What are your dietary preferences in your daily life? What foods do you like to eat and what foods do you not eat? Why?
Question 10	Do your appetite and food intake change after you become malnourished? What are the changes?

#### 2.1.2 Two‐Round Delphi Method

Two rounds of Delphi expert consultation were conducted via email between May to July 2023. A panel of 15 experts was purposefully selected based on the following inclusion criteria: (a) holding a senior professional title or higher, (b) having > 5 years of clinical experience in renal disease and nutrition and (c) possessing a Bachelor's degree or higher. The experts were required to provide their feedback within a 2 week timeframe for each round. The reliability of the expert consultation was evaluated using two indices:

- The expert positive coefficient (response rate) was assessed, with a rate exceeding 70% deemed satisfactory (Zhang et al. 2022).
- The authority coefficient (Cr) was calculated as the mean of judgement basis (Ca) and the degree of familiarity (Cs), where a Cr ≥ 0.7 indicated acceptable expert authority (Guan et al. 2019).

#### 2.1.3 Screening of Items

The relevance and clarity of the items were evaluated using the boundary value method, which incorporated the full score frequency, arithmetic mean and coefficient of variation (Wang et al. 2021). The boundary threshold for both frequency and mean was set at ‘mean−standard deviation’ (with items scoring above this threshold being retained). Conversely, the threshold for the coefficient of variation was set at ‘mean + standard deviation’ (with items scoring below this threshold being retained).

### 2.2 Test of Validity and Reliability

#### 2.2.1 Pilot Study

Prior to the formal survey, a pilot study employing convenience sampling was conducted with 20 CKD patients (Hertzog 2008). The purpose of the pilot study was to enable the research team to review ambiguous or impractical phrasing, thereby ensuring clarity and field applicability.

#### 2.2.2 Participants

Reliability and validity testing was conducted from July to November 2023. The total sample was split into two independent subsamples for exploratory factor analysis (EFA) and confirmatory factor analysis (CFA) to ensure structural stability and facilitate cross‐validation (Costello and Osborne 2005).

For the EFA subsample, the sample size was determined based on a participant‐to‐item ratio of 5:1 (Costello and Osborne 2005) and a minimum of 150 cases. Accounting for a 10% dropout rate, 152 participants were recruited. For the CFA subsample, 151 participants were recruited, which is generally considered adequate for models with well‐defined factor structures and moderate‐to‐high factor loadings (Muthen 2003; Wolf et al. 2013). Strong communalities and factor loadings, as observed in our study, further compensate for the modest sample size (MacCallum et al. 1999).

Participants were required to meet the following inclusion criteria: (a) a diagnosis of CKD, (b) aged 18 years or older, (c) admission to a nephrology ward and (d) provision of voluntary informed consent. Patients with concurrent severe psychiatric disorders that could impede cooperation were excluded.

#### 2.2.3 Data Collection

Standardized guidelines were used to explain the purpose of the survey to eligible inpatients, emphasizing confidentiality and anonymity. Participants completed the questionnaires independently. Upon completion, researchers conducted an immediate on‐site review to identify and rectify any missing responses or logical inconsistencies. The verified questionnaires were subsequently coded for data analysis.

### 2.3 Data Analysis

Statistical analyses were performed using SPSS version 26.0 and Mplus version 7.3. Content validity was evaluated using the content validity index, with an acceptable threshold of CVI ≥ 0.83 (Lynn 1986). For the dichotomous Perceived Knowledge (K) domain, item analysis was conducted using the difficulty index (ideal range: 0.31–0.60) (Tarrant et al. 2009) and the discrimination index (acceptable threshold: > 0.20) (Kumar et al. 2021). Here, the difficulty index was interpreted as the proportion of participants endorsing that they knew the content of the item and the discrimination index reflected the item's ability to differentiate participants with higher versus lower overall perceived malnutrition knowledge.

EFA was performed using principal‐axis factoring with oblique rotation. Item retention was based on eigenvalues greater than 1, factor loadings ≥ 0.50 and communalities ≥ 0.25 (Kline 2023). During CFA, the general loading threshold was ≥ 0.50; however, marginal loadings (> 0.40) were retained if theoretically essential and without adverse effects on model fit or reliability (Hair et al. 2010). For CFA, the maximum likelihood robust (MLR) estimator was applied to the Attitude and Practice domains due to multivariate non‐normality (Mardia's test *p* < 0.05), while the weighted least squares mean and variance adjusted (WLSMV) estimator was utilized for the dichotomous Perceived Knowledge domain. Model fit indices were considered acceptable if the SRMR and RMSEA were ≤ 0.08 and the CFI and TLI were ≥ 0.90 (Kline 2023). Convergent validity was established if the composite reliability (CR) was ≥ 0.70 and the average variance extracted (AVE) was > 0.50 (Raykov and Marcoulides 2015). Discriminant validity was confirmed if inter‐factor correlations were ≤ 0.85 (Brown 2015) and the square root of the AVE for each construct exceeded its bivariate correlations with other constructs (Fornell and Larcker 1981). Internal consistency reliability was deemed appropriate when Cronbach's alpha exceeded 0.60 (Cho and Kim 2015).

### 2.4 Ethical Considerations

The study was conducted in accordance with the Declaration of Helsinki and approved by the Human Ethical Committee of the Universiti Sains Malaysia (code: USM/JEPeM/22070470) and the Ethics Committee of the Shaanxi Provincial Hospital of Traditional Chinese Medicine (code: 2022067).

## 3 Results

### 3.1 Generation of Items Based on Previous Qualitative Findings

As detailed in our previous publication (Zhu, Gao, et al. 2024), content analysis of the semi‐structured interviews yielded three primary domains (Perceived Knowledge, Attitude and Practice) and seven subcategories. Rather than reproducing the exhaustive qualitative codes here, this study focused on adapting those findings to generate the initial item pool for the Malnutrition KAP Scale.

Specifically, items within the Perceived Knowledge domain were drafted to assess patients' basic understanding of CKD‐related malnutrition, while also addressing common misconceptions and cultural dietary influences. For the Attitude domain, items were formulated to capture both negative psychological barriers (e.g., lack of attention, external dependence) and positive motivators (e.g., self‐awareness, desire to learn). Finally, items in the Practice domain were designed to evaluate daily behaviours, contrasting passive or disadvantageous habits with favourable nutritional management strategies (e.g., adhering to a high‐protein, balanced diet). This structured translation of published qualitative themes yielded the preliminary 38‐item pool.

### 3.2 Expert Characteristics

The Delphi panel comprised 15 experts: Ten nephrology specialists, three clinical nutrition specialists and two experts with extensive experience in psychometrics and scale development. These experts represented eight institutions across five regions in two countries. The panel was highly experienced, with over 80% having more than 10 years of clinical or research experience and 75% holding an associate senior professional title or higher. The response rate was 89.5% in the first round and 93.75% in the second round. The authority coefficient (Cr) for this study was 0.77, indicating a high degree of expert authority and reliability.

### 3.3 Screening of Items

Following two rounds of Delphi consultation, 11 items were removed due to failing to meet predefined statistical thresholds or based on expert consensus. This process resulted in a preliminary 27‐item scale. A comprehensive summary of the item reduction process is provided in Table 2.

**TABLE 2:** Summary of the item reduction process from initial pool to final scale.

Stage of reduction	Number of items removed	Specific items removed (item code & content)	Primary reason for deletion
Delphi process: Round 1	7	K7: Do you know that regional food cultures can affect nutritional status? K8: Do you know that religious food culture can affect nutritional status? A3: You believe that relying entirely on the arrangements of others would be detrimental to the management of nutrition. P4: You choose a high protein diet in your daily life to improve your disease state. P8: Has your diet changed since the onset of the disease? P9: You go to the hospital for regular nutritional screenings. P12: You find a balance between culture or religion and diet in nutritional management.	Failed to meet the established boundary values for all three predefined criteria: full score frequency, arithmetic mean and coefficient of variation.
Delphi process: Round 2	4	K8: Do you know how to evaluate the nutritional status of CKD patients? K10: Do you know that oral supplementation is a good replacement to ordinary food? K7: Do you know how malnutrition affects the health status of people with CKD? K9: Do you know the dietary principles to prevent malnutrition in patients with chronic kidney disease?	K8 & K10: Failed to meet the specified standards for full score frequency, arithmetic mean and coefficient of variation. K7 & K9: Exhibited discrepancies in one or two indicators; removed after comprehensive discretion by the research team to optimize scale precision.
Item analysis	1	K1: Do you know malnutrition?	Displayed undesirable Difficulty Index (DIF I) and Discrimination Index (DI) compared to the acceptable thresholds (DIF I = 0.53, DI = 0.36, which were deemed insufficient relative to other retained items).
Confirmator factor analysis (CFA)	1	K5: Do you know how malnutrition and chronic renal disease are related?	Exhibited the lowest factor loading (0.304), which is below the acceptable threshold. The item was removed to improve model fit.
Final scale	Total removed: 13	Initial pool of 38 items−13 removed items = 25 items in the final scale	

*Note:* Item codes (e.g., K7, K8) represent the internal numbering used during that specific stage of analysis. Items were renumbered iteratively after each phase of reduction.

### 3.4 Pilot Study

Prior to formal psychometric testing, a pilot study was conducted with 20 CKD patients to assess the clarity, readability, comprehensibility and acceptability of the preliminary scale. The response rate was 100%. All participants reported that the items were clear and easy to understand, with completion times ranging from 8 to 14 min. The content was unanimously deemed relevant and acceptable and no difficulties or suggestions for revision were reported. Consequently, no modifications were made to the instrument and all 27 items were retained for the formal validation phase.

### 3.5 Validity and Reliability

#### 3.5.1 Participant Characteristics

For the formal psychometric evaluation, 330 questionnaires were distributed. Of these, 319 were returned (response rate: 96.67%) and 303 complete questionnaires (91.82%) were deemed valid for final analysis. The sample was randomly split into two subsets: 152 participants for EFA and 151 for CFA. Demographically, the sample predominantly comprised males (*n* = 193, 63.70%) and individuals of Han nationality (*n* = 291, 96.03%). The mean age was 48.26 ± 13.78 years. The majority of participants resided in urban areas (*n* = 184, 60.70%) and were married (*n* = 214, 70.60%). Further demographic details are provided in Table 3.

**TABLE 3:** Demographic characteristics of the participants (*n* = 303).

	Participants	Frequency (*n*)	Percentage (%)	Mean (SD)
Gender	Male	193	63.70	
	Female	110	36.30	
Age (years)				48.26 (13.78)
Nationality	The Han nationality	291	96.03	
	Other ethnic groups	12	3.97	
Place of residence	City	184	60.70	
	Village	119	39.30	
Marital status	Single	45	14.90	
	Married	214	70.60	
	Seperated/Divorced	26	8.60	
	Widowed	18	5.90	
Education level	Postgraduate students and above	75	24.80	
	Undergraduate	93	30.70	
	Junior college or high school	75	24.80	
	Secondary school and below	60	19.80	
Occupation	Worker	45	14.85	
	Farmer	86	28.38	
	Civil servant	37	12.21	
	Professional	53	17.49	
	Unemployed	43	14.19	
	Self‐employed (housewife)	39	12.87	
Per capita monthly household income	≥ 5001 Yuan	101	33.30	
	3001–5000 Yuan	141	46.50	
	≤ 3000 Yuan	61	20.10	
CKD staging	Stage 1	58	19.14	
	Stage 2	52	17.16	
	Stage 3a	55	18.15	
	Stage 3b	29	9.57	
	Stage 4	12	3.96	
	Stage 5	97	32.02	
Course of the disease	≤ 1 year	76	25.09	
	1–3 years	73	24.09	
	4–5 years	56	18.48	
	6–10 years	52	17.16	
	> 10 years	46	15.18	
Presence of co‐morbidity	Nil	136	44.90	
	Yes	167	55.10	
Dialysis or not	Nil	173	57.10	
	Yes	130	42.90	

#### 3.5.2 Validity

##### 3.5.2.1 Content Validity

Content validity was evaluated using item‐level (I‐CVI) and scale‐level (S‐CVI) indices. The I‐CVI ranged from 0.93 to 1.00 for Perceived Knowledge and from 0.93 to 1.00 for both Attitude and Practice, all exceeding the recommended threshold of 0.83. Furthermore, the S‐CVI/Ave was 0.99 across all three domains (acceptable criterion > 0.90). The S‐CVI/UA values for Perceived Knowledge, Attitude and Practice were 0.88, 0.89 and 0.90, respectively, demonstrating robust content validity. These results confirm that the items were highly relevant, representative and comprehensive (File S1).

##### 3.5.2.2 Item Analysis of Perceived Knowledge Dimension

Among the eight Perceived Knowledge items, seven exhibited optimal difficulty indices (DIF I: 0.31–0.60) and excellent discrimination indices (DI ≥ 0.25). Notably, no item displayed a negative DI. However, one item (K1) exhibited suboptimal DIF I and DI and was consequently deleted (Table 4). Following the removal of K1, seven items remained in the Perceived Knowledge domain. To maintain continuity, the remaining items were renumbered consecutively (e.g., the original K2 became the new K1).

**TABLE 4:** Distribution of items in relation to DIF I, DI and action recommended in perceived knowledge items.

Parameter (Range)	Interpretation	Items (*N* = 8)	Action
Difficulty index			
≤ 30	Difficult	0 (0%)	Revise/Discard
31–40	Good	1 (12.5%)	Store in question bank
41–60	Excellent	6 (75.0%)	Store in question bank
≥ 61	Easy	1 (12.5%)	Revise/Discard
Discrimination Index			
< 0.20	Poor	1 (12.5%)	Revise/Discard
0.21–0.24	Acceptable	0 (0%)	Store in question bank
0.25–0.34	Good	4 (50.0%)	Store in question bank
≥ 0.35	Excellent	3 (37.5%)	Store in question bank

##### 3.5.2.3 Exploratory Factor Analysis (EFA)

Construct validity for the ‘Attitude’ and ‘Practice’ domains was initially evaluated using EFA.

For the attitude domain, the Kaiser–Mayer–Olkin (KMO) measure was 0.931 and Bartlett's test of sphericity was significant (*x* ^2^ = 1105.738, *p* < 0.01). A single‐factor solution emerged (eigenvalue > 1), explaining 69.87% of the total variance, which was supported by the scree plot (Figure 1). All item factor loadings exceeded 0.50 (Table 5).

**FIGURE 1:**
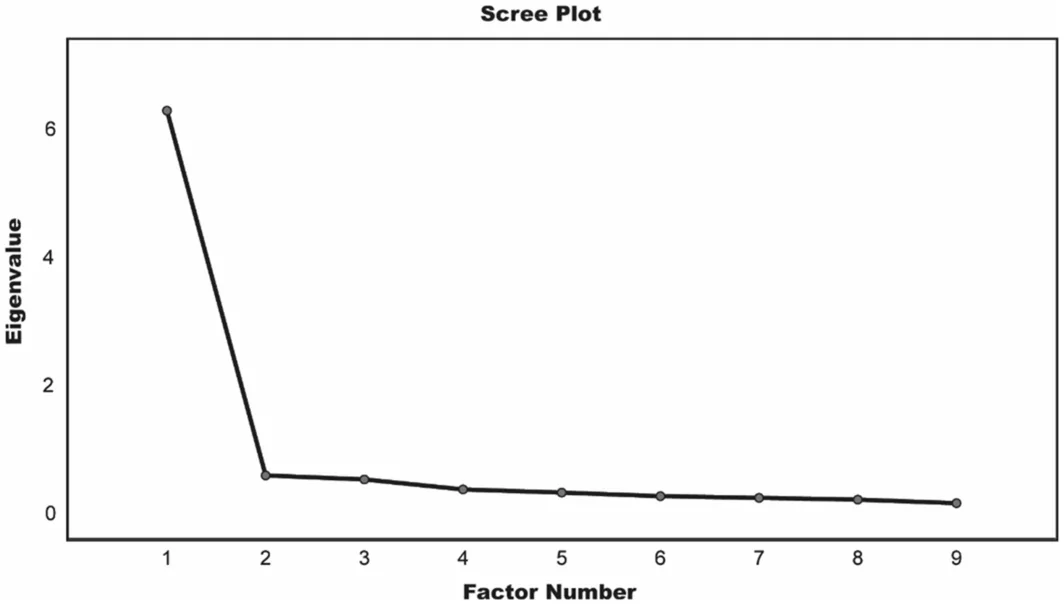
Scree plot showing the one factor above the point of inflexion.

**TABLE 5:** Exploratory factor loading of attitude items.

Items content	Factor loading
A1: CKD patients need to have a comprehensive understanding of the importance of nutrition.	0.888
A2: Fostering optimistic beliefs and attitudes plays a vital role in promoting the adoption of healthy behaviours in the context of nutrition management.	0.861
A3: Having adequate knowledge on malnutrition will assist me to have a better quality of life and lower the risk of complications.	0.857
A4: Preventing malnutrition is crucial to support the treatment and prognosis of CKD.	0.853
A5: Improving self‐control could assist in nutrition management of CKD.	0.847
A6: My nutritional status has an impact on the development of CKD.	0.839
A7: Diet therapy is a part of important treatment regiments for CKD.	0.819
A8: Nutritional management can help delay the disease process.	0.804
A9: Nutrition counselling is important for CKD patients.	0.748

*Note:* Principal Axis Factoring was used as the extraction method. Promax oblique rotation with Kaiser normalization was used as the rotation method. Values of factor loading < 0.5 are suppressed and not displayed.

Similarly, for the practice dimension, the KMO measure was 0.906 and Bartlett's test of sphericity was significant (*x* ^2^ = 999.681, *p* < 0.01). One factor was extracted, explaining 61.84% of the total variance, consistent with the scree plot (Figure 2). All factor loadings were > 0.50 (Table 6). Following item analysis and EFA, the preliminary structure comprised 7 items for perceived knowledge, 9 items for attitude and 10 items for practice.

**FIGURE 2:**
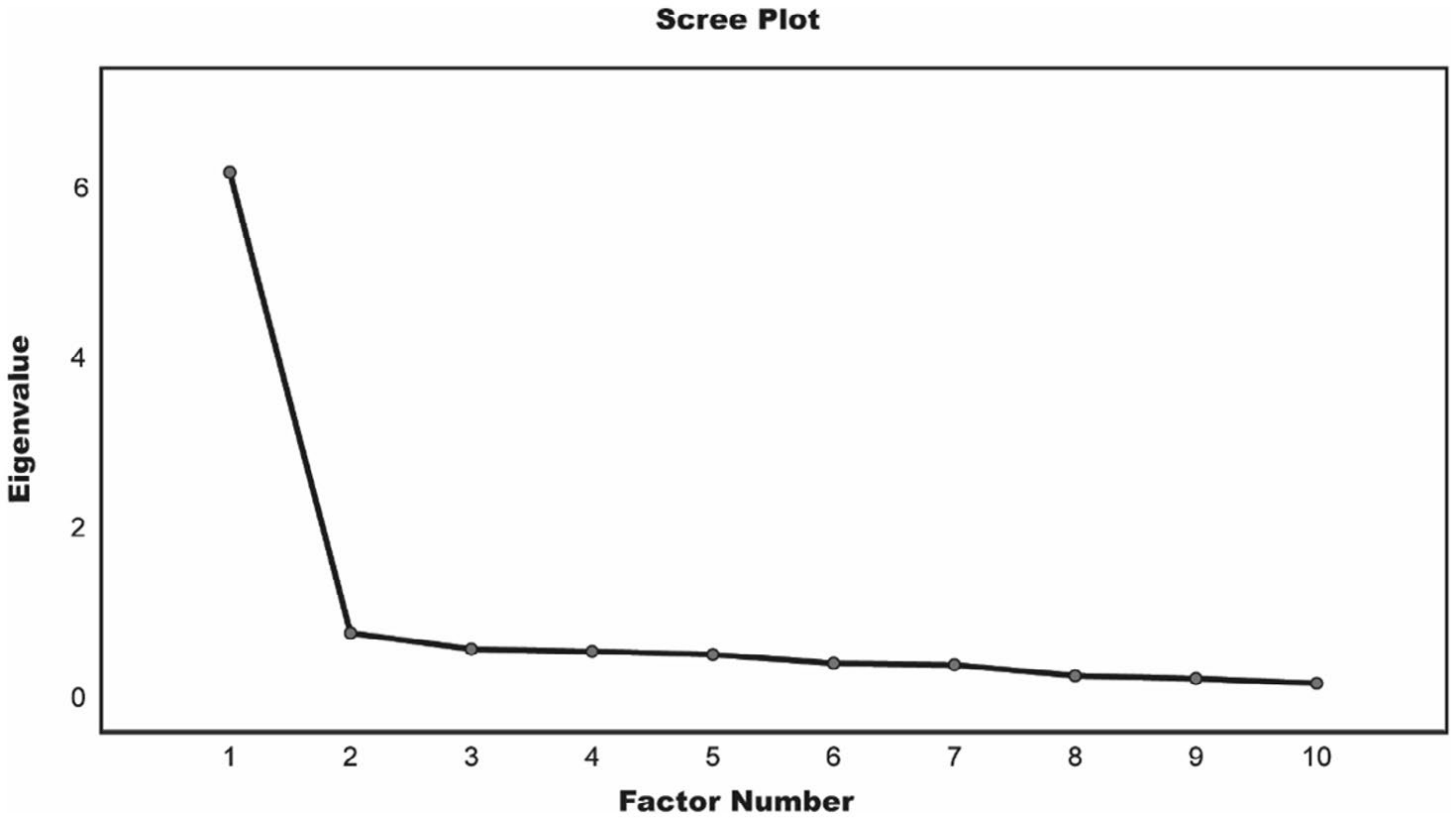
Scree plot showing the one factor above the point of inflexion.

**TABLE 6:** Exploratory factor loading of practice items.

Items content	Factor loading
P1: I give priority to my nutrition intake.	0.859
P2: I am proactive in adopting the right approach to manage my nutritional status.	0.850
P3: I gained knowledge about nutrition for CKD patients from various sources.	0.792
P4: I measure my weight regularly to screen for malnutrition.	0.789
P5: I consume high quality protein diet daily to improve my nutrition status.	0.788
P6: I will learn about renal nutrition so that I can consume an appropriate diet.	0.781
P7: I change my poor eating habits to a healthier one.	0.771
P8: I am flexible in changing my diet since the onset of the disease is depending on my condition and knowledge.	0.759
P9: I received regular dietary counselling and education.	0.744
P10: I adhere to the dietary recommendations given by my doctor/dietician.	0.720

*Note:* Principal axis factoring was used as the extraction method. Promax oblique rotation with Kaiser normalization was used as the rotation method. Values of factor loading < 0.5 are suppressed and not displayed.

##### 3.5.2.4 Confirmatory Factor Analysis (CFA)

Due to violations of the multivariate normality assumption across all models, the weighted least squares mean and variance adjusted (WLSMV) estimator was applied to the Perceived Knowledge model (categorical data), while the robust maximum likelihood (MLR) estimator was utilized for the Attitude and Practice models.

The initial Perceived Knowledge model (Model‐1) produced a poor fit to the data (Table 7). Item K5, which displayed an insufficient factor loading of 0.304, was removed. Removing item K5 yielded Model‐2, which fit the data well (CFI = 0.978, TLI = 0.963, SRMR = 0.061, RMSEA (90% CI) = 0.070 (< 0.001, 0.124), RMSEA *p*‐value = 0.248). Standardized item loadings ranged from 0.488 to 0.806 (Table 8). Although one retained item exhibited a loading of 0.488—marginally below the traditional 0.50 threshold—it was kept in the final model. This decision was based on its extremely close proximity to the 0.50 cut‐off, its indispensable theoretical significance to the integrity of the Perceived Knowledge domain and the fact that the overall composite reliability (CR = 0.743) and model fit remained robust.

**TABLE 7:** Model fit indices for two models of Perceived Knowledge Model.

Model	CFI	TLI	SRMR	RMSEA (90% CI)	RMSEA *p*
Model‐1 (Initial)	0.957	0.935	0.080	0.080 (0.033, 0.124)	0.128
Model‐2^a^	0.978	0.963	0.061	0.070 (0.000–0.127)	0.248

Abbreviations: CFI, comparative fit index; CI, confidence interval; Cl fit, close fit; RMSEA, root mean square error of approximation; SRMR, standardized root mean square residual; TLI, Tucker–Lewis index.

^a^ Measurement model with K5 was removed.

**TABLE 8:** Standardized item loading for model‐2 of perceived knowledge model.

Items	Model‐1	Model‐2	CR (95% CI)	AVE
	Standardized item loading			
K1: Do you know your nutritional status?	0.815	0.806	0.743 (0.680–0.806)	0.520
K2: Do you know the symptoms of malnutrition?	0.781	0.788		
K3: Do you know the risk factors of malnutrition?	0.515	0.488		
K4: Do you aware the consequences of malnutrition to CKD patients?	0.659	0.653		
K5: Do you know how malnutrition and chronic renal disease are related?	0.304	—		
K6: Do you know the daily protein intake requirement for CKD patients?	0.722	0.744		
K7: Do you know the preventive measures towards malnutrition among CKD patients?	0.758	0.759		

*Note:* AVE was calculated as the average of the squared standardized item loadings. An AVE > 0.50 and CR ≥ 0.70 indicate acceptable convergent validity.

Abbreviations: AVE, average variance extracted; CR, composite reliability.

For the Attitude domain, although the initial loadings (Model‐1) exceeded 0.50, the overall fit was inadequate (Table 9). The model was iteratively modified based on modification indices and strong theoretical justifications via post hoc correlated residuals. We sequentially correlated the residuals of items A9 and A8 (Model‐2; shared focus on professional nutritional interventions), A3 and A1 (Model‐3; shared emphasis on cognitive nutrition perceived knowledge), A7 and A9 (Model‐4) and A8 and A7 (Model‐5). The correlations among A7, A8 and A9 are conceptually justified as they all relate to formal therapeutic interventions. The final model (Model‐5) achieved an acceptable fit: CFI = 0.982, TLI = 0.972, SRMR = 0.019, RMSEA = 0.077 (90% CI: 0.040–0.111, *p* = 0.104). Loadings ranged from 0.827 to 0.914 (Table 10), with an excellent CR of 0.968.

**TABLE 9:** Model fit indices for five models of attitude model.

Model^a^	CFI	TLI	SRMR	RMSEA (90% CI)	RMSEA *p*
Model‐1 (Initial)	0.940	0.920	0.029	0.129 (0.102–0.158)	< 0.001
Model‐2^a^	0.961	0.947	0.025	0.106 (0.077–0.136)	0.002
Model‐3^b^	0.969	0.955	0.023	0.097 (0.066–0.129)	0.008
Model‐4^c^	0.974	0.961	0.022	0.090 (0.058–0.123)	0.090
Model‐5^d^	0.982	0.972	0.019	0.077 (0.040–0.111)	0.104

Abbreviations: CFI, comparative fit index; CI, confidence interval; Cl fit, close fit; RMSEA, root mean square error of approximation; SRMR, standardized root mean square residual; TLI, Tucker–Lewis index.

^a^ Correlated residual for item A9 with A8.

^b^ Correlated residual for item A3 with A1.

^c^ Correlated residual for item A7 with A9.

^d^ Correlated residual for item A8 with A7.

**TABLE 10:** Standardized item loading for model‐5 of Attitude Model.

Items	Model‐1	Model‐2	Model‐3	Model‐4	Model‐5	CR (95% CI)	AVE
	Standardized item loading						
A1	0.881	0.890	0.878	0.881	0.889	0.968 (0.960–0.977)	0.776
A2	0.902	0.910	0.904	0.906	0.914		
A3	0.876	0.880	0.868	0.870	0.874		
A4	0.886	0.892	0.891	0.893	0.897		
A5	0.893	0.896	0.897	0.897	0.897		
A6	0.883	0.881	0.886	0.886	0.880		
A7	0.915	0.908	0.915	0.910	0.897		
A8	0.878	0.861	0.865	0.864	0.848		
A9	0.863	0.845	0.850	0.846	0.827		

*Note:* AVE was calculated as the average of the squared standardized item loadings. An AVE > 0.50 and CR ≥ 0.70 indicate acceptable convergent validity.

Abbreviations: AVE, average variance extracted; CR, composite reliability.

The initial measurement model for the Practice domain (Model‐1) displayed a poor fit to the data (Table 11). Based on theoretical rationale, residual correlations were added between P2 and P1 (Model‐2; representing proactive prioritization of nutrition), P9 and P8 (Model‐3; representing perceived knowledge‐guided dietary adjustments) and P7 and P4 (Model‐4; focusing on concrete self‐monitoring behaviours). The final model (Model‐4) yielded a strong fit: CFI = 0.976, TLI = 0.967, SRMR = 0.027, RMSEA = 0.071 (90% CI: 0.039–0.101, *p* = 0.122). All factor loadings for Model‐4 exceeded 0.50 (Table 12) and the CR was 0.957.

**TABLE 11:** Model fit indices for five models of practice model.

Model^a^	CFI	TLI	SRMR	RMSEA (90% CI)	RMSEA *p*
Model‐1 (Initial)	0.939	0.922	0.034	0.109 (0.084–0.135)	< 0.001
Model‐2^a^	0.951	0.935	0.032	0.100 (0.073–0.126)	0.002
Model‐3^b^	0.962	0.948	0.029	0.089 (0.061–0.117)	0.013
Model‐4^c^	0.976	0.967	0.027	0.071 (0.039–0.101)	0.122

Abbreviations: CFI, comparative fit index; CI, confidence interval; Cl fit, close fit; RMSEA, root mean square error of approximation; SRMR, standardized root mean square residual; TLI, Tucker–Lewis index.

^a^ Correlated residual for item P2 with P1.

^b^ Correlated residual for item P9 with P8.

^c^ Correlated residual for item P7 with P4.

**TABLE 12:** Standardized item loading for model‐5 of Practice Model.

Items	Model‐1	Model‐2	Model‐3	Model‐4	CR (95% CI)	AVE
	Standardized item loading					
P1	0.839	0.823	0.825	0.824	0.957 (0.945–0.968)	0.694
P2	0.836	0.820	0.822	0.820		
P3	0.855	0.856	0.860	0.857		
P4	0.883	0.884	0.888	0.902		
P5	0.832	0.837	0.834	0.831		
P6	0.869	0.876	0.879	0.879		
P7	0.818	0.817	0.818	0.840		
P8	0.837	0.842	0.830	0.824		
P9	0.762	0.765	0.748	0.737		
P10	0.817	0.811	0.810	0.806		

*Note:* AVE was calculated as the average of the squared standardized item loadings. An AVE > 0.50 and CR ≥ 0.70 indicate acceptable convergent validity.

Abbreviations: AVE, average variance extracted; CR, composite reliability.

Discriminant validity was established using the Fornell‐Larcker criterion. The square roots of the Average Variance Extracted (AVE) for Perceived Knowledge, Attitude and Practice were 0.721, 0.881 and 0.833, respectively. The inter‐construct correlations ranged from 0.443 to 0.585. Since the square root of the AVE for each dimension was strictly greater than all corresponding off‐diagonal correlations, satisfactory discriminant validity was confirmed.

##### 3.5.2.5 Final Scale and Scoring System

Following the removal of K5 during CFA, the final Malnutrition KAP Scale comprised 25 items across three dimensions. The Perceived Knowledge dimension included 6 items, with each item scored as 1 point for a ‘yes’ response and 0 points for a ‘no’ response, yielding a possible score range of 0–6. The Attitude dimension included 9 items assessed using a 5‐point Likert scale: strongly disagree = 1, disagree = 2, not sure = 3, agree = 4 and strongly agree = 5, with a possible score range of 9–45. The Practice dimension included 10 items assessed using a 5‐point Likert scale: never = 1, occasionally = 2, sometimes = 3, often = 4 and always = 5, with a possible score range of 10–50. Subscale scores were calculated by summing the item scores within each dimension. Because Perceived Knowledge, Attitude and Practice represent distinct constructs measured using different response formats, domain scores are reported and interpreted separately rather than combined into a total score. Higher scores in each respective domain indicate greater perceived knowledge of malnutrition‐related content, more positive attitudes and better practices among patients with CKD. No items were reverse‐scored. The final instrument and scoring instructions are provided in File S2.

#### 3.5.3 Reliability

The domain‐specific Cronbach's alphas were 0.740 (Perceived Knowledge), 0.970 (Attitude) and 0.958 (Practice). No further improvement in the Cronbach's alpha values could be achieved by deleting any of the items, indicating good internal consistency within each domain of the scale.

## 4 Discussion

In this present study, we developed and validated the 25‐item CKD Malnutrition KAP (CKD‐MKAP) scale. To the best of our knowledge, this is the first instrument specifically designed to assess malnutrition‐related KAP among Chinese patients with CKD. Given that a significant proportion of malnourished Chinese CKD patients receive inadequate nutritional education (Jiang and Li 2021), this scale facilitates early detection and proactive management by systematically evaluating their perceived knowledge, attitudes and practices (Dai et al. 2022).

Grounded in the KAP theory (Nwagbara et al. 2021), the scale comprises three interconnected domains. For the Perceived Knowledge domain (designed as a quiz of all type‐A questions), classical test theory parameters—Difficulty Index (DIF I) and Discrimination Index (DI)—were employed to guide item selection. Because this domain was constructed as a dichotomous self‐assessment of whether respondents perceived themselves as knowing each malnutrition‐related concept, the ‘difficulty index’ in this study represents endorsement rate rather than objectively demonstrated correctness. With the exception of item K1, all perceived knowledge items fell within the optimal DIF I range (0.31–0.60) and exhibited satisfactory DI (≥ 0.20), indicating appropriate difficulty and excellent discriminative ability (Hassan and Hod 2017; Kumar et al. 2021). Notably, no negative DI were observed, ruling out issues such as item ambiguity or incorrect scoring keys (Hassan et al. 2016). Consequently, K1 was removed. The retained items demonstrated excellent content validity, with I‐CVI values ranging from 0.93 to 1.00 (Lynn 1986).

For construct validity, EFA extracted a single common factor for both the Attitude and Practice domains, explaining 69.87% and 61.84% of the variance, respectively (factor loadings > 0.5), aligning perfectly with the theoretical KAP framework (Swisher et al. 2004). EFA explores the underlying dimensionality to determine the most suitable factor structure for the scale, whereas CFA complements this by verifying the identified factor structure and its corresponding items (Koohi et al. 2021). During this phase, item K5 showed a low factor loading and was excluded, which significantly enhanced model fit. As its conceptual essence was already captured by item K4, the elimination of item K5 compromised neither the scale's theoretical framework nor its overall content. The finalized models—comprising Perceived Knowledge (6 items), Attitude (9 items) and Practice (10 items)—demonstrated acceptable fit indices (RMSEA, SRMR, CFI and TLI) after necessary model modifications.

In this study, internal consistency was evaluated using two reliability metrics: Cronbach's alpha and composite reliability (CR), following the formula proposed by Raykov and Marcoulides (Raykov and Marcoulides 2015). The scale exhibited excellent internal consistency within each domain, with Cronbach's alphas of 0.740, 0.970 and 0.958 for Perceived Knowledge, Attitude and Practice, respectively (Cho and Kim 2015). To address the potential limitations of coefficient alpha in CFA studies, we calculated CR to provide a less biased estimate (Hox et al. 2010; Lim et al. 2016). The CR values for the KAP models were 0.743, 0.968 and 0.957, respectively, ensuring good structural reliability.

Compared to other renal KAP instruments, the CKD‐MKAP scale demonstrates robust psychometric properties despite its brevity. For instance, a recently developed 77‐item nutritional KAP questionnaire for non‐dialysis CKD Stage 3–5 patients reported acceptable domain Cronbach's alpha values of 0.861, 0.816 and 0.612, respectively (Mahapatra et al. 2026). Our 25‐item scale is significantly more concise—thereby reducing respondent burden in clinical settings—yet achieves comparable or superior reliability. The slightly lower alpha in our Perceived Knowledge domain (0.740) remains acceptable and is likely attributable to the limited number of dichotomously scored items. Furthermore, regarding validity, dietary management and phosphorus control KAP scales developed for haemodialysis patients have reported CVI values ranging from 0.79 to 0.99 and cumulative variances in EFA between 64.21% and 74.52% (Zhang et al. 2023; Zhu, Mohd Yusoff, et al. 2024; Wu et al. 2025). The CKD‐MKAP scale showed similarly strong validity, with I‐CVI values of 0.93–1.00 and construct validity confirmed by both EFA and CFA. Despite differences in target populations, item formats and measurement purposes, these comparisons suggest that the CKD‐MKAP scale is a concise and psychometrically robust tool for assessing malnutrition‐related KAP among Chinese patients with CKD.

Clinically, the CKD‐MKAP scale serves as a practical tool for identifying gaps in patients' nutritional understanding, beliefs and self‐management behaviours, helping healthcare professionals tailor targeted nutrition education. In research settings, it holds potential to function as a reliable outcome measure for evaluating educational interventions in the future. However, because internal consistency and structural validity do not establish responsiveness to change, additional measurement‐property testing—including evaluations of temporal stability and responsiveness—must be conducted before the scale can be formally adopted for this specific purpose. Specifically, higher Perceived Knowledge scores reflect a better understanding of malnutrition‐related concepts, risk factors, prevention and management; higher Attitude scores indicate more positive beliefs, awareness and willingness to engage in malnutrition prevention; and higher Practice scores reflect more frequent and appropriate self‐management behaviours.

A major strength of this study lies in its rigorous, theoretically grounded methodology (Al‐Qerem et al. 2022). The scale's development integrated literature reviews, semi‐structured interviews and a two‐round Delphi process, followed by comprehensive psychometric validation. Importantly, the items were culturally adapted to reflect Chinese dietary habits, values and linguistic nuances. By providing a multidimensional assessment, this instrument equips clinicians and nurses to better manage CKD‐related malnutrition, ultimately aiming to improve patient outcomes and quality of life.

## 5 Limitations

Several limitations of this study should be acknowledged. First, the sample for the reliability and validity testing of the scale was recruited from a single city in Northwest China, which may limit the generalizability of the findings and potentially compromise the stability of the statistical model. Future studies should conduct cross‐regional and cross‐cultural validations to ensure the scale's applicability across diverse populations and settings. Second, the temporal stability of the instrument remains unknown because test–retest reliability was not assessed. Additionally, the scale's responsiveness to change has not yet been evaluated. Therefore, its application as an outcome measure to evaluate educational interventions remains a potential future application that requires additional longitudinal psychometric validation. Third, although the sample sizes for EFA and CFA met traditional guidelines and demonstrated psychometric adequacy, a formal a priori power analysis was not conducted. Future validation studies should employ rigorous power estimation methods to confirm the stability of the factor structure. Fourth, the final CFA models for the Attitude and Practice domains required several post hoc correlated residuals to achieve acceptable fit. Because these data‐driven modifications were not independently cross‐validated, the correlations among A7, A8 and A9 may reflect local dependence or an additional Attitude subdimension, which warrants further investigation in future large‐scale studies.

## 6 Implications for Nursing Practice

The CKD‐MKAP scale has important practical implications for clinical nursing. This concise, 25‐item instrument is easy to administer and suitable for routine use by ward nurses, outpatient staff and other healthcare professionals without substantially increasing their workload. By assessing perceived knowledge, attitudes and practices separately, it helps identify specific barriers to optimal nutritional status and supports targeted interventions; for example, patients with adequate perceived knowledge but poor practices may benefit more from behavioural counselling than informational brochures. The validated scale can also provide a standardized metric for nursing quality improvement and for evaluating dietary education programs over time. Its integration into routine nursing assessments may ultimately promote more precise, individualized and effective nutritional care for patients with CKD.

## 7 Conclusion

In conclusion, the CKD‐MKAP scale was developed through a rigorous methodological framework and demonstrated robust validity and reliability within the current sample. It can serve as a valuable instrument for healthcare professionals to assess malnutrition‐related perceived knowledge, attitudes and practices among Chinese patients with CKD in comparable clinical settings, thereby facilitating the identification of areas requiring targeted educational interventions. However, given that the study was confined to a single region in China, further multicenter and cross‐cultural validation studies are warranted before the scale can be generalized to broader populations.

## Author Contributions

**Shengrui Zhu:** data curation, investigation, conceptualization, methodology, validation, writing – original draft, writing – review and editing, formal analysis, software. **Hafzan Yusoff:** supervision, methodology, conceptualization, writing – review and editing. **Kueh Yee Cheng:** supervision, methodology, software. **Dariah Mohd Yusoff:** supervision, conceptualization, methodology. **Xiujuan Feng:** data curation, investigation. **Wen‐Kai Zheng:** investigation. **Hongfang Chen:** investigation.

## Funding

The authors have nothing to report.

## Disclosure

The authors have nothing to report.

## Conflicts of Interest

The authors declare no conflicts of interest.

## Supporting information


**File S1:** Table 1. The relevance ratings on the perceived knowledge dimension.
Table 2. The relevance ratings on the attitude dimension.
Table 3. The relevance ratings on the practice dimension.

**File S2:** Table 1. Perceived Knowledge related to malnutrition.
Table 2. Attitude related to malnutrition.
Table 3. Practice related to malnutrition.

## Data Availability

The data that support the findings of this study are available on request from the corresponding author. The data are not publicly available due to privacy or ethical restrictions.
