# Supplementary material for: Development and Validation of CKD‐MKAP: A Malnutrition Perceived Knowledge, Attitude, and Practice (KAP) Scale for Patients With Chronic Kidney Diseases (CKD) in China
Source: Nurs Open. 2026 Oct 9;13(10):e70925. doi: 10.1002/nop2.70925 (PMC13652517; doi:10.1002/nop2.70925)
Supplement: Supplementary file 1 [file NOP2-13-e70925-s001.docx]

**Development and Validation of CKD-MKAP: A Malnutrition Perceived Knowledge, Attitude, and Practice (KAP) Scale for Patients with Chronic Kidney Diseases (CKD) in China**

**Scoring Instructions for the Malnutrition KAP Scale:**

The Malnutrition KAP Scale comprised 25 items across three dimensions. The Perceived Knowledge dimension included 6 items, with each item scored as 1 point for a “yes” response and 0 points for a “no” response, yielding a possible score range of 0–6. The Attitude dimension included 9 items assessed using a 5-point Likert scale: strongly disagree = 1, disagree = 2, not sure = 3, agree = 4, and strongly agree = 5, with a possible score range of 9–45. The Practice dimension included 10 items assessed using a 5-point Likert scale: never = 1, occasionally = 2, sometimes = 3, often = 4, and always = 5, with a possible score range of 10–50. Subscale scores were calculated by summing the item scores within each dimension. Higher scores in each respective domain indicate greater perceived knowledge of malnutrition-related content, more positive attitudes, and better practices among patients with CKD. No items were reverse-scored.

**SECTION 1: Perceived Knowledge related to Malnutrition in CKD patient**

Please tick [√] the statement that relate to your real situation.

**Table 1: Perceived Knowledge related to Malnutrition**

| **Item** | **Level of perceived knowledge** | |
| --- | --- | --- |
|  | **No** | **Yes** |
| 1. Do you know your nutritional status? |  |  |
| 2. Do you know the symptoms of malnutrition? |  |  |
| 3. Do you know the risk factors of malnutrition? |  |  |
| 4. Do you aware the consequences of malnutrition to CKD patients? |  |  |
| 5. Do you know the daily protein intake requirement for CKD patients? |  |  |
| 6. Do you know the preventive measures towards malnutrition among CKD patients? |  |  |

**SECTION 2: Attitude related to Malnutrition in CKD patient**

Please tick [√] the statement that relate to your real situation.

**Table 2: Attitude related to malnutrition**

| **Item** | **Level of agreement** | | | | |
| --- | --- | --- | --- | --- | --- |
|  | **1** | **2** | **3** | **4** | **5** |
|  | **Strongly disagree** | **Disagree** | **Not sure** | **Agree** | **Strongly agree** |
| 1. CKD patients need to have a comprehensive understanding of the importance of nutrition. |  |  |  |  |  |
| 2. Fostering optimistic beliefs and attitudes plays a vital role in promoting the adoption of healthy behaviors in the context of nutrition management. |  |  |  |  |  |
| 3. Having adequate knowledge on malnutrition will assist me to have a better quality of life, and lower the risk of complications. |  |  |  |  |  |
| 4. Preventing malnutrition is crucial to support the treatment and prognosis of CKD. |  |  |  |  |  |
| 5. Improving self-control could assist in nutrition management of CKD. |  |  |  |  |  |
| 6. My nutritional status has an impact on the development of CKD. |  |  |  |  |  |
| 7. Diet therapy is a part of important treatment regiments for CKD. |  |  |  |  |  |
| 8. Nutritional management can help delay the disease process. |  |  |  |  |  |
| 9. Nutrition counseling is important for CKD patients. |  |  |  |  |  |

**SECTION 3: Practice related to Malnutrition in CKD patient**

Please tick [√] the statement that relate to your real situation.

**Table 3: Practice related to Malnutrition**

| **Item** | **Frequency of occurrence** | | | | |
| --- | --- | --- | --- | --- | --- |
|  | **1** | **2** | **3** | **4** | **5** |
|  | **Never** | **Occasi-onally** | **Sometimes** | **Often** | **Always** |
| 1. I give priority to my nutrition intake. |  |  |  |  |  |
| 2. I am proactive in adopting the right approach to manage my nutritional status. |  |  |  |  |  |
| 3. I gained knowledge about nutrition for CKD patients from various sources. |  |  |  |  |  |
| 4. I measure my weight regularly to screen for malnutrition. |  |  |  |  |  |
| 5. I consume high quality protein diet daily to improve my nutrition status. |  |  |  |  |  |
| 6. I will learn about renal nutrition so that I can consume an appropriate diet. |  |  |  |  |  |
| 7. I change my poor eating habits to a healthier one. |  |  |  |  |  |
| 8. I am flexible in changing my diet since the onset of the disease is depending on my condition and knowledge. |  |  |  |  |  |
| 9. I received regular dietary counselling and education. |  |  |  |  |  |
| 10. I adhere to the dietary recommendations given by my doctor/dietician. |  |  |  |  |  |
