# Supplementary material for: Development and Validation of CKD‐MKAP: A Malnutrition Perceived Knowledge, Attitude, and Practice (KAP) Scale for Patients With Chronic Kidney Diseases (CKD) in China
Source: Nurs Open. 2026 Oct 9;13(10):e70925. doi: 10.1002/nop2.70925 (PMC13652517; doi:10.1002/nop2.70925)
Supplement: Supplementary file 2 [file NOP2-13-e70925-s002.docx]

With regards to the content validity, 15 experts with high academic standards in the fields of nephrology, clinical nutrition, and scale development were consulted. Each item was rated on a scale of 1-4. The researcher calculated Item Content Validity Index (I-CVI), Universal Agreement (UA), Scale-level Content Validity Index based on the Average method (S-CVI/Ave) and Scale-level Content Validity Index based on the Universal Agreement method (S-CVI/UA) separately.

For the perceived knowledge dimension, the study's results indicated that the I-CVI scores for each item ranged from 0.99 to 1.00, all surpassing the 0.83 threshold. The S-CVI/Ave score was 0.99, which was greater than 0.90. The UA scores ranged from 0 to 1, resulting in an S-CVI/UA of 0.88, again surpassing the 0.83 standard. These findings collectively suggest that all item CVI values were deemed valid, signifying that the scale items better reflected the research topic (see Supplementary Table 1).

**Table 1 The relevance ratings on the perceived knowledge dimension**

| **Item** | **Expert in Agreement** | **I-CVI** | **UA** |
| --- | --- | --- | --- |
| K1 | 15 | 1 | 1 |
| K2 | 15 | 1 | 1 |
| K3 | 15 | 1 | 1 |
| K4 | 15 | 1 | 1 |
| K5 | 15 | 1 | 1 |
| K6 | 14 | 0.93 | 0 |
| K7 | 15 | 1 | 1 |
| K8 | 15 | 1 | 1 |
|  | **S-CVI/Ave** | 0.99 |  |
|  | **S-CVI/UA** |  | 0.88 |

Use N for expert, I-CVI: the expert in agreement divided by the number of experts; UA: score ‘1’ is assigned to the item that achieved 100% experts in agreement, while score ‘0’ because not all the experts provided relevance rating of 1; S-CVI/Ave: the average of I-CVI scores across all items; S-CVI/UA: the average of UA scores across all items.

The study's findings for the attitude dimension revealed that the I-CVI scores for each item ranged from 0.93 to 1.00, resulting S-CVI/Ave score was 0.99. The UA scores ranged from 0 to 1, resulting in an S-CVI/UA of 0.89. All value surpassing the 0.83 standard (refer to Supplementary Table 2).

**Table 2 The relevance ratings on the attitude dimension**

| **Item** | **Expert in Agreement** | **I-CVI** | **UA** |
| --- | --- | --- | --- |
| A1 | 15 | 1 | 1 |
| A2 | 14 | 0.93 | 0 |
| A3 | 15 | 1 | 1 |
| A4 | 15 | 1 | 1 |
| A5 | 15 | 1 | 1 |
| A6 | 15 | 1 | 1 |
| A7 | 15 | 1 | 1 |
| A8 | 15 | 1 | 1 |
| A9 | 15 | 1 | 1 |
|  | **S-CVI/Ave** | 0.99 |  |
|  | **S-CVI/UA** |  | 0.89 |

Use N for expert, I-CVI: the expert in agreement divided by the number of experts; UA: score ‘1’ is assigned to the item that achieved 100% experts in agreement, while score ‘0’ because not all the experts provided relevance rating of 1; S-CVI/Ave: the average of I-CVI scores across all items; S-CVI/UA: the average of UA scores across all items.

The study's findings for the practice dimension revealed that the I-CVI scores for each item ranged from 0.93 to 1.00. The S-CVI/Ave score was 0.99, which was greater than 0.90. The UA scores ranged from 0 to 1, resulting in an S-CVI/UA of 0.9, again surpassing the 0.83 standard (refer to Supplementary Table 3).

**Table 3 The relevance ratings on the practice dimension**

| **Item** | **Expert in Agreement** | **I-CVI** | **UA** |
| --- | --- | --- | --- |
| P1 | 15 | 1 | 1 |
| P2 | 15 | 1 | 1 |
| P3 | 15 | 1 | 1 |
| P4 | 15 | 1 | 1 |
| P5 | 14 | 0.93 | 0 |
| P6 | 15 | 1 | 1 |
| P7 | 15 | 1 | 1 |
| P8 | 15 | 1 | 1 |
| P9 | 15 | 1 | 1 |
| P10 | 15 | 1 | 1 |
|  | **S-CVI/Ave** | 0.99 |  |
|  | **S-CVI/UA** |  | 0.9 |

Use N for expert, I-CVI: the expert in agreement divided by the number of experts; UA: score ‘1’ is assigned to the item that achieved 100% experts in agreement, while score ‘0’ because not all the experts provided relevance rating of 1; S-CVI/Ave: the average of I-CVI scores across all items; S-CVI/UA: the average of UA scores across all items.
